# Conventional Agar-Based Culture Method, and Nucleic Acid Amplification Test (NAAT) of the cppB Gene for Detection of Neisseria gonorrhea in Pregnant Women Endocervical Swab Specimens

**DOI:** 10.5812/ircmj.3726

**Published:** 2013-03-05

**Authors:** Parvin Hassanzadeh, Jalal Mardaneh, Mohammad Motamedifar

**Affiliations:** 1 Department of Biology, School of Sciences, Shiraz University, Shiraz, IR Iran; 2 Department of Pathobiology, School of Public Health and Institute of Public Health Research, Tehran University of Medical Sciences, Tehran, IR Iran; 3 Alborzi Clinical Microbiology Research Center, Shiraz University of Medical Sciences, Shiraz, IR Iran; 4 Department of Bacteriology & Virology, Medical School, Shiraz HIV/Aids Research Center

**Keywords:** Pregnant Women, Neisseria Gonorrhea, Culture, Nucleic Acid Amplification Techniques (NAAT)

## Abstract

**Background:**

Neisseria gonorrhea is the etiological agent of the sexually transmitted disease (STD) gonorrhea, and primarily infects the mucous membranes of the urethra, endocervix, pharynx or rectum of females which may result in substantial morbidity. N. gonorrhea also causes disseminated infection, with complications that may result in ectopic pregnancy, tubal infertility, chronic pelvic pain or maternal transmission of gonorrhea, and also increases susceptibility to HIV.

**Objectives:**

In the present investigation, we used conventional agar-based culture method, and nucleic acid amplification of CCPB gene for detection of Neisseria gonorrhea in endocervical swabs samples collected from pregnant women studied

**Patients and Methods:**

Endocervical swabs specimens for this study were obtained from 1100 pregnant women who presented to Shiraz (Iran) Hospitals from 2009 to 2011. In the present investigation we used conventional agar-based culture method, and nucleic acid amplification test (NAAT) of CCPB gene for detection of Neisseria gonorrhea in endocervical swabs samples collected from pregnant women studied. From each pregnant woman two endocervical swabs were taken: one swab placed in tubes containing phosphate buffered saline for Polymerase Chain Reaction, and the other to inoculate on culture media.

**Results:**

Among 1100 endocervical swabs examined, 13 (1.18%) samples had positive results by polymerase chain reaction (PCR) on Neisseria gonorrhea CCPB gene. All endocervical swabs culture had negative results for Neisseria gonorrhea. 84 (7%) of the women had vaginal discharge, in whom PCR on endocervical swabs of these individuals had negative findings.

**Conclusions:**

Nucleic acid amplification tests (NAATs) are very appropriate in detection of infected individuals. Detection techniques such as NAATs are independent of bacterial viability, and have a potential to limit false negative samples, therefore, in our country, the application of different laboratory diagnosis methods including NAATs with culture as gold standard for determination antimicrobial susceptibility is essential.

## 1. Background

Neisseria gonorrhea is the etiological agent of the sexually transmitted disease (STD) gonorrhea. Approximately 62 million new cases of gonococcal disease occur annually worldwide. N. gonorrhea primarily infects the mucous membranes of the urethra, endocervix, pharynx or rectum of females which may result in substantial morbidity. N. gonorrhea also causes disseminated infection, with complications that may result in ectopic pregnancy, tubal infertility, chronic pelvic pain or maternal transmission of gonorrhea, and also increases susceptibility to HIV. Gonorrhea primarily causes urethritis in males (1-3). Health complications resulting from gonorrhea disease occur mainly in women, and are largely assigned to the predominately asymptomatic nature of the lower genital tract infection. Untreated, subclinical infection of the cervix can lead to upper genital tract involvement and, potentially, to infertility (4, 5). Similar to Chlamydia trachomatis, 80% of women (though only 10% of men) infected with Neisseria gonorrhea are asymptomatic, and the most common and serious complications of the infection are also infertility, ectopic pregnancy, and pelvic inflammatory disease (5, 6). A number of laboratory methods have been advanced to diagnosis genital infections caused by *N. gonorrhea*. Conventional diagnosis techniques of gonorrhea require culture on selective media or an observation of intracellular gram-negative diplococcic in smears prepared of urethral or endocervical swabs. Despite the low viability of the *N. gonorrhea* in vitro, the current gold standard for diagnosis of infections caused by this organism is culture on selective media, because culture gives us the opportunity to determinate antimicrobial susceptibility testing, and continuous monitoring of antibiotic resistance profile is crucial for appropriate management of cases as resistance could vary in different regions and over different time periods. However, even under optimal laboratory conditions, the sensitivity of *N. gonorrhea* cultures ranges from 85% to 95% for acute infection, and falls to approximately 50% for females with chronic infections. This is largely due to bacterial autolysis, poor sampling techniques, and improper specimen storage and transport. Nucleic acid amplification tests (NAATs), have been shown to have both high sensitivity and specificity for the detection of *N. gonorrhea*(1, 7-10). Detection techniques such as NAATs which are independent of bacterial viability have a potential to limit false negative samples and in some geographic areas, NAATs are promptly substituting the culture method for the diagnosis of gonorrhea disease (8). The growing health burden of STDs and their spiraling costs have led to a need for rapid and reliable laboratory techniques to identify the causative pathogens (11).

## 2. Objectives

In the present investigation we used conventional agar-based culture method and nucleic acid amplification of CCPB gene for detection of Neisseria gonorrhea in endocervical swabs samples collected from pregnant women studied.

## 3. Materials and Methods 

### 3.1. Study Population

Endocervical swabs specimens for this study were obtained from 1100 pregnant women who presented to Shiraz (Iran) Hospitals from 2009 to 2011. Before sample collection, all study population were registered with a code number.

### 3.2. Sampling

Expert nurses used Dacron-tipped swabs for specimen collection from pregnant women endocervix. From each pregnant woman two endocervical swabs were taken: one swab placed in screw-cap tubes containing phosphate buffered saline (PBS) at -200C for Polymerase Chain Reaction (PCR), and the other to inoculate on culture media. The samples were sent to the Microbiology Laboratory for diagnostic processing.

### 3.3. Microbiological Studies

In laboratory endocervical swabs were inoculated on nonselective chocolate agar and selective Modified Thayer Martin (MTM) medium plate containing Vancomycin (3 µg/ml), Colistin (7.5 µg/ml), Nystatin (12.5 units/ml), and Amphotericin B (5 µg/ml). Smear was prepared for gram staining. Cultures plates were incubated at 35°C ± 0.5 in candle-flam extinction jar (for CO_2_), and incubated for 48 h. Colonies of N. gonorrhea were identified by using standard diagnostic tests including colony morphology (raised, 0.5 to 1 mm in diameter, transparent, glistening pearly white or dew-drop like), gram staining (gram negative, typically coccoid shaped, arranged in pairs (diplococcic) resembling coffee beans ), oxidase test (positive), superoxol test (positive), carbohydrate degradation test (utilizes only glucose).

### 3.4. Molecular Studies

The endocervical swabs maintained for PCR identification were brought to room temperature, and then vortexed for one minute to release the material contained in the swab. The swabs were then thrown away and the suspension was centrifuged for five minutes at 3000 rpm to pellet the cells. After removing the supernatant by aspiration, the cells were suspended in 100 mL of K-buffer (1x PCR buffer with 0.5% nonionic detergent Tween-20 and 200 µL /mL Proteinase K). The cell suspension was incubated at 55 ºC for one hour, and then heated to 95ºC for 10 minutes to inactivate the Proteinase K. The sequence data on the cppB gene carried on chromosome as well as on 4.2 kb cryptic plasmid of N. gonorrhea was used to select two 20-mer oligonucleotide primers as designated below: sense 5׳-GCT ACG CAT ACC CGC GTT GC-3׳ and antisense 5׳-CGA AGA CCT TCG AGC AGA CA-3׳.

The expected length of the amplified product of the target sequence with these primers was 390 bp. Amplification was performed in 25 µL reaction volume containing: 1 µL of each of the primers (100ng/µl), 1 µL dNTPs (10 mM), 2.5 µL 1x PCR buffer, 2.5 µL MgCl2 (50 mM) in 11.8 µL deionized water, 5 µL template DNA, and finally 0.2 µL Taq DNA polymerase. All the reagents were taken in a 0.5 mL PCR tube, and mixed by gentle vortexing before overlaying with a drop of mineral oil. Thirty five cycles of amplification were performed in a DNA thermal cycler. Each PCR cycling reaction consisted of the followings: denaturation at 94ºC for 4 minutes followed by denaturation at 94ºC for 60 seconds, annealing at 48ºC for 60 seconds, amplification at 72ºC for 60 seconds, and final extension at 72 ºC for 10 minutes. The PCR product bands of 390 bp were separated by electrophoresis through a 1.5% (w/v) agarose gel in 1x TAE buffer. The DNA bands were visualized by ethidium bromide staining, and using a UV transilluminator, and the results were documented.

## 4. Results

**Figure 1.**
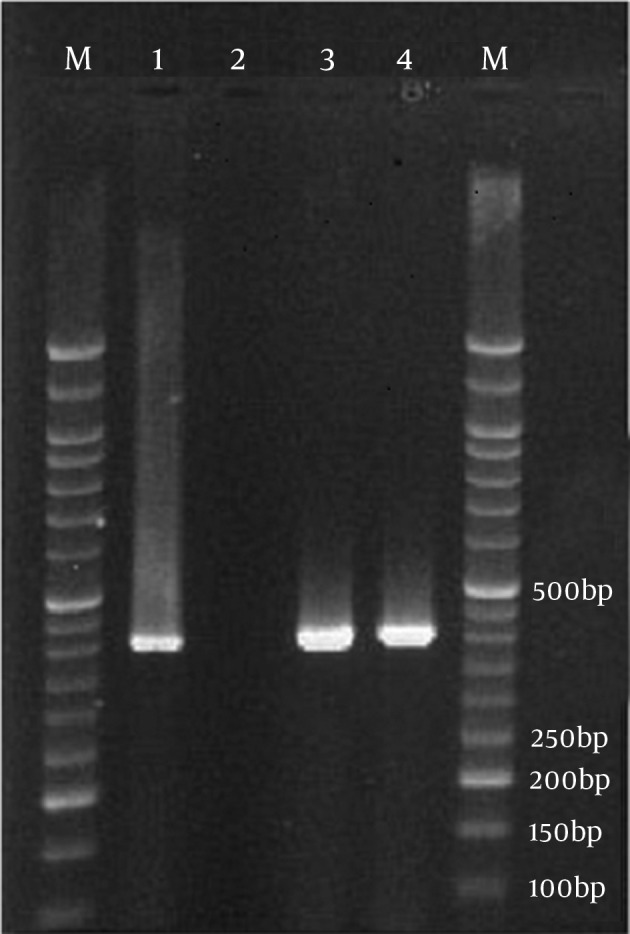
Identiﬁcation ofNeisseria gonorrhea in Endocervical Swabs Specimens After PCR Ampliﬁcation of ccpB Gene Speciﬁc Primers M, molecular weight marker (50 bp ladder);Lane 1, positive control; Lane 2, negative control;Lanes 3, 4, positive samples.

Among 1100 endocervical swabs examined, 13 (1.18%) samples had positive results by polymerase chain reaction (PCR) on Neisseria gonorrhea CCPB gene. All endocervical swabs culture had negative results for Neisseria gonorrhea. Eighty four (7.6%) of the women had vaginal discharge that PCR results on endocervical swabs of these individuals were negative. In addition, none of the patients had known conditions associated with acquired immunodeﬁciency, underlying diseases or antibiotic therapy. The profile of contraceptive methods that used by pregnant women included in this study is summarized in Table 1. As shown in Figure 1, the PCR demonstrated ccpB gene ampliﬁed fragment (about 390 bp) that was speciﬁc for Neisseria gonorrhea.

**Table 1.** Contraceptive Methods Usedby Pregnant Women in Multiple Pregnancies

Contraceptive by use	Multiple Pregnancies			
	First Pregnancy (n = 774), No. (%)	Second Pregnancy (n = 220), No. (%)	Third Pregnancy (n = 70), No. (%)	Fourth Pregnancy (n= 36), No. (%)
**Oral Contraceptive Pill (OCP)**	182 (23.5)	63 (28.5)	30 (43)	10 (28)
**Condom**	237 (30.5)	94 (43)	15 (21)	4 (11)
**Intrauterine Device (IUD)**	0	16 (7)	5 (7 )	4 (11)
**Natural Methods**	355 (46)	47 (21.5)	20 (29)	18 (50)

## 5. Discussion

Sexually transmitted diseases (STDs) are a broad but relatively well-defined group of infections, usually described by acute presentations which can be developed to a chronic clinical condition. Both men and women are affected. STDs constitute a hidden epidemic of enormous physical, psychological, and economic consequences (4, 5, 12). Our data revealed that gonococcal infections could occur for asymptomatic women who had not any risk factor, and its incidence is low. We applied conventional agar-based method combination with nucleic acid amplification assay for detection of *Neisseria gonorrhea* in this research. Results showed that NAATs are very useful in detection of infected individuals, and is a noninvasive technology for detecting prevalent gonorrhea. Detection techniques such as NAATs are independent of bacterial viability, and have a potential to limit false negative samples, therefore, in our country, the application of different laboratory diagnosis methods including NAATs with culture as gold standard for determining antimicrobial susceptibility is essential. On the other hand the augment resistance to current antibiotics that are used in gonococcal treatment is concerning. It seems that one of its reasons is the erratically use of these antibiotics. Therefore communication between clinic and laboratory is needed to ensure optimal treatment of infection. Progresses in the laboratory methods in the diagnosis of common sexually transmitted pathogens may supply an opportunity to objectively quantify sexual risk behaviors of young people. For isolation of the fastidious bacteria including *N. gonorrhea*, optimal sampling, transport media, transport time, and transport conditions are necessary for executing culture diagnostics with high sensitivity and specificity (8). Rapid diagnosis and treatment of STIs in female reduce complications and sequel, assist the prevention of further transmission of bacteria to healthy individuals, and avoid paying additional medical costs, and reduce defects and disasters which may result from untreated infections, incomplete or inappropriate treatment. Religion, civil legislation, prejudice, stereotyping, social stigma, and shame play effective roles in reducing sexually transmitted diseases (STDs) such as gonorrhea in Iran. Religiosity and spirituality supply an important coping framework in the lives of many individuals in Iran. Greater religious involvement in Iran is a protective factor for STD risk by intensifying self-efficacy to communicate with partners about sex, STDs, and pregnancy; self-efficacy to deny unsafe sex; positive sentiments towards condom use; and the delay of sexual debut. In addition, the rate of risk factors including social and economic factors such as poverty, unemployment, low levels of education, drug and alcohol use, poor personal hygiene, and having multiple sex partners is relatively very low in Shiraz city, where the study was conducted. Although commercially available tests established upon nucleic acid amplification have greater sensitivity than conventional tests for detecting N. gonorrhea, specificity remains controversial, since intra- and interspecies genetic recombination take place frequently between members of the genus Neisseria. Cross-reactivity is common with most target sequences, including the 16S rRNA gene, and false-positive results with *Neisseria meningitides*, and *Neisseria lactamase* have been reported using cppB gene sequences (1). However, each of these tests has limitations, including variable sensitivities to inhibitors, cross-reactivity with other microorganisms, limited sensitivity, high costs, and equipment of public health laboratories. In addition, their application is often restricted to specific specimen types due to limited validation of the assays (10, 13) cppB gene and 16S rRNA gene-based assays are used for confirmation, however, about 5% of *N. gonorrhea* strains do not carry the cppB plasmid, and not all 16S rRNA-based tests are sensitive and specific enough (1, 7). This can be a disadvantage in the use of this cppB gene for detection of *Neisseria gonorrhea*. People who procured sexually transmitted diseases (STDs) seemed to be more susceptible to HIV acquisition and transmission. STDs may also raise the expression of HIV binding ligands which can expedite HIV acquisition and transmission. Although ulcerative STDs have been associated with the highest rates of enhancing HIV susceptibility, the presence of inflammatory STDs, such as Neisseria gonorrhea and Chlamydia trachomatis, also result in the recruitment of inflammatory cells, and potentiate HIV acquisition and transmission (2, 14). Increasingly, public health guidelines should focus on wide-spread screening of women for Neisseria gonorrhea and other strategies, such as expedited partner therapy, to break the cycle of heterosexual STI transmission and reinfection. By focusing on the high risk sexual dyad, rather than the individual diagnosed with an STI, it is likely that a more comprehensive treatment and prevention effort would occur (15, 16). The condition is complicated by the decreasing antibiotic susceptibility of the common sexually transmitted bacteria such as *Neisseria gonorrhea* to commonly used antibiotics, especially the penicillin group (17). The World Health Organization therefore established in 1990 a surveillance program in nine regions of the world (the Gonococcal Antimicrobial Surveillance Program, or GASP) (13, 18, 19). Unfortunately to date no data on gonococcal susceptibility in Iran are available. With attention expanding concerns about decreasing treatment options for gonorrhea disease, preserving the efficacy of currently used treatment regimen, and ensuring best Neisseria gonorrhea antibiotic resistance surveillance are of the extreme influential (13, 17, 19-23). Antibiotics approved by the CDC usually obliterate Neisseria gonorrhea, but reinfection is common between patients, this suggesting that gonococcal infection in humans may be unsuccessful to draw out long-lasting protective immunity, at least in some patients with low levels of serum and genital mucosal anti gonococcal antibodies after infection. Genetic factors, such as cytokine gene polymorphisms and/or human leukocyte antigen (HLA) alleles, may be responsible for variable immune system responses to Neisseria gonorrhea. Cytokine gene polymorphisms can regulate cytokine secretion subsequent to activation of immune system, whereas, HLA alleles participate to the specificity of T-cell responses to *Neisseria thgonorrheath *(20).The Centers for Disease Control and Prevention recommend routine screening for sexually transmitted infections at the first prenatal visit, and third trimester repeat screening, is advised for women under the age of 25, or at raised infection risk. Proactive screening and basic treatment of these common cervical infections, especially among those infected with HIV-1 and HSV-2, should be considered for young sexually active women in Communities with a high incidence of HIV/STIs (24, 25).

## References

[1] Mayta H, Calderon M, Taverna J, Montenegro S, Balqui J, Campos K (2006). Use of a reliable PCR assay for the detection of Neisseria gonorrhoeae in Peruvian patients.. Clin Microbiol Infect..

[2] Mhlongo S, Magooa P, Muller EE, Nel N, Radebe F, Wasserman E (2010). Etiology and STI/HIV coinfections among patients with urethral and vaginal discharge syndromes in South Africa.. Sex Transm Dis..

[3] Whiley DM, Tapsall JW, Sloots TP (2006). Nucleic acid amplification testing for Neisseria gonorrhoeae: an ongoing challenge.. J Mol Diagn..

[4] Edwards JL, Butler EK (2011). The Pathobiology of Neisseria gonorrhoeae Lower Female Genital Tract Infection.. Front Microbiol..

[5] Forhan SE, Gottlieb SL, Sternberg MR, Xu F, Datta SD, McQuillan GM (2009). Prevalence of sexually transmitted infections among female adolescents aged 14 to 19 in the United States.. Pediatrics..

[6] Franceschi S, Smith JS, van den Brule A, Herrero R, Arslan A, Anh PT (2007). Cervical infection with Chlamydia trachomatis and Neisseria gonorrhoeae in women from ten areas in four continents. A cross-sectional study.. Sex Transm Dis..

[7] Geraats-Peters CW, Brouwers M, Schneeberger PM, van der Zanden AG, Bruisten SM, Weers-Pothoff G (2005). Specific and sensitive detection of Neisseria gonorrhoeae in clinical specimens by real-time PCR.. J Clin Microbiol..

[8] Hjelmevoll SO, Olsen ME, Sollid JU, Haaheim H, Melby KK, Moi H (2008). Clinical validation of a real-time polymerase chain reaction detection of Neisseria gonorrheae porA pseudogene versus culture techniques.. Sex Transm Dis..

[9] Nizamuddin S, Jabeen K, Zafar A (2011). Evaluation of predominant Neisseria gonorrhoeae strain types and its correlation with fluoroquinolone resistance in Pakistan.. J Pak Med Assoc..

[10] Schachter J, Moncada J, Liska S, Shayevich C, Klausner JD (2008). Nucleic acid amplification tests in the diagnosis of chlamydial and gonococcal infections of the oropharynx and rectum in men who have sex with men.. Sex Transm Dis..

[11] Lee SR, Chung JM, Kim YG (2007). Rapid one step detection of pathogenic bacteria in urine with sexually transmitted disease (STD) and prostatitis patient by multiplex PCR assay (mPCR).. J Microbiol..

[12] Faber MT, Nielsen A, Nygard M, Sparen P, Tryggvadottir L, Hansen BT (2011). Genital chlamydia, genital herpes, Trichomonas vaginalis and gonorrhea prevalence, and risk factors among nearly 70,000 randomly selected women in 4 Nordic countries.. Sex Transm Dis..

[13] Dicker LW, Mosure DJ, Steece R, Stone KM (2004). Laboratory tests used in US public health laboratories for sexually transmitted diseases, 2000.. Sex Transm Dis..

[14] Mayer KH, Venkatesh KK (2011). Interactions of HIV, other sexually transmitted diseases, and genital tract inflammation facilitating local pathogen transmission and acquisition.. Am J Reprod Immunol..

[15] Detels R, Green AM, Klausner JD, Katzenstein D, Gaydos C, Handsfield H (2011). The incidence and correlates of symptomatic and asymptomatic Chlamydia trachomatis and Neisseria gonorrhoeae infections in selected populations in five countries.. Sex Transm Dis..

[16] Thurman AR, Holden AE, Shain RN, Perdue ST (2009). The male sexual partners of adult versus teen women with sexually transmitted infections.. Sex Transm Dis..

[17] Fayemiwo SA, Muller EE, Gumede L, Lewis DA (2011). Plasmid-mediated penicillin and tetracycline resistance among Neisseria gonorrhoeae isolates in South Africa: prevalence, detection and typing using a novel molecular assay.. Sex Transm Dis..

[18] Dorlencourt F, Boireaux C, Sednaoui P, Danilenko NV, Legros D (2002). In vitro susceptibility of 120 strains of Neisseria gonorrhoeae isolated in Kyrghyzstan.. Sex Transm Dis..

[19] Ohnishi M, Golparian D, Shimuta K, Saika T, Hoshina S, Iwasaku K (2011). Is Neisseria gonorrhoeae initiating a future era of untreatable gonorrhea?: detailed characterization of the first strain with high-level resistance to ceftriaxone.. Antimicrob Agents Chemother..

[20] Geisler WM, Wang C, Tang J, Wilson CM, Crowley-Nowick PA, Kaslow RA (2008). Immunogenetic correlates of Neisseria gonorrhoeae infection in adolescents.. Sex Transm Dis..

[21] Goire N, Freeman K, Tapsall JW, Lambert SB, Nissen MD, Sloots TP (2011). Enhancing gonococcal antimicrobial resistance surveillance: a real-time PCR assay for detection of penicillinase-producing Neisseria gonorrhoeae by use of noncultured clinical samples.. J Clin Microbiol..

[22] Stupiansky NW, Van Der Pol B, Williams JA, Weaver B, Taylor SE, Fortenberry JD (2011). The natural history of incident gonococcal infection in adolescent women.. Sex Transm Dis..

[23] Yuan LF, Yin YP, Dai XQ, Pearline RV, Xiang Z, Unemo M (2011). Resistance to azithromycin of Neisseria gonorrhoeae isolates from 2 cities in China.. Sex Transm Dis..

[24] Berggren EK, Patchen L (2011). Prevalence of Chlamydia trachomatis and Neisseria gonorrhoeae and repeat infection among pregnant urban adolescents.. Sex Transm Dis..

[25] Venkatesh KK, van der Straten A, Mayer KH, Blanchard K, Ramjee G, Lurie MN (2011). African women recently infected with HIV-1 and HSV-2 have increased risk of acquiring Neisseria gonorrhoeae and Chlamydia trachomatis in the Methods for Improving Reproductive Health in Africa trial.. Sex Transm Dis..

